# Modulation of Heterochromatin by Male Specific Lethal Proteins and *roX* RNA in *Drosophila melanogaster* Males

**DOI:** 10.1371/journal.pone.0140259

**Published:** 2015-10-15

**Authors:** S. Kiran Koya, Victoria H. Meller

**Affiliations:** Department of Biological Sciences, Wayne State University, Detroit, Michigan, United States of America; University of Maryland School of Medicine, UNITED STATES

## Abstract

The ribonucleoprotein Male Specific Lethal (MSL) complex is required for X chromosome dosage compensation in *Drosophila melanogaster* males. Beginning at 3 h of development the MSL complex binds transcribed X-linked genes and modifies chromatin. A subset of MSL complex proteins, including MSL1 and MSL3, is also necessary for full expression of autosomal heterochromatic genes in males, but not females. Loss of the non-coding *roX* RNAs, essential components of the MSL complex, lowers the expression of heterochromatic genes and suppresses position effect variegation (PEV) only in males, revealing a sex-limited disruption of heterochromatin. To explore the molecular basis of this observation we examined additional proteins that participate in compensation and found that MLE, but not Jil-1 kinase, contributes to heterochromatic gene expression. To determine if identical regions of *roX* RNA are required for dosage compensation and heterochromatic silencing, we tested a panel of *roX1* transgenes and deletions and find that the X chromosome and heterochromatin functions are separable by some mutations. Chromatin immunoprecipitation of staged embryos revealed widespread autosomal binding of MSL3 before and after localization of the MSL complex to the X chromosome at 3 h AEL. Autosomal MSL3 binding was dependent on MSL1, supporting the idea that a subset of MSL proteins associates with chromatin throughout the genome during early development. The broad localization of these proteins early in embryogenesis supports the idea of direct action at autosomal sites. We postulate that this may contribute to the sex-specific differences in heterochromatin that we, and others, have noted.

## Introduction

Heterochromatin, which comprises one third of the *Drosophila melanogaster* genome, makes up half of the X chromosome, most of the 4^th^ chromosome and the entire Y chromosome. Although primarily composed of repetitive non-coding DNA, hundreds of genes are embedded in heterochromatic regions. Expression of these genes is thought to require mechanisms that overcome the repressive chromatin environment, and, interestingly, these genes also require the heterochromatic environment for optimal expression [1, 2]. The structure of heterochromatin is generally thought to be independent of sex, but some differences in male and female heterochromatin have been detected. Conditional depletion of Heterochromatic Protein 1 (HP1), a major component of heterochromatin, causes preferential gene misregulation and lethality in males [3]. These authors also found the distribution of HP1 to be slightly different in males and females. In accord with these observations, our laboratory discovered that full expression of autosomal heterochromatic genes in males requires factors that are unnecessary in females [4]. In brief, loss of both *roX* RNAs, redundant members of Male Specific Lethal (MSL) complex, reduced expression of autosomal heterochromatic genes in males, but not females [4]. Partial loss of *roX* function allows escaper males with strong suppression of Position Effect Variegation (PEV) of autosomal heterochromatic reporters in males, but no suppression of PEV is observed in *roX* mutant females [4, 5]. These two observations reveal a sex-based difference in heterochromatin. Interestingly, the intact MSL complex is not required for the heterochromatic function as loss of Male Specific Lethal 2 (MSL2), an essential member of MSL complex, had no effect on expression of heterochromatic genes or PEV [4, 5]. These findings rule out indirect effects of dosage compensation failure, such as relocalization of X chromosome-bound factors or reduced expression of an X-linked protein essential for heterochromatin formation. Two additional MSL proteins, Male Specific Lethal 1 and 3 (MSL1, MSL3) are also necessary for full expression of autosomal heterochromatic genes in males [4]. Taken together, these findings indicate differences in autosomal heterochromatin in the sexes that are revealed by loss of a subset of the molecules in the MSL complex. This may reflect differences in the establishment or maintenance of heterochromatin, or the sensitivity of heterochromatin to loss of specific molecules.

In this study we examined *roX1* mutants to determine whether the dosage compensation and heterochromatin functions of *roX1* are separable. We find that maintenance of PEV is achieved at a much lower level of *roX1* RNA than is required for dosage compensation, and identify *roX1* mutations that differentially affect these processes. We extend previous studies by demonstrating the involvement of MLE, but not Jil-1, in the expression of heterochromatic genes in males. To explore the possibility that a subset of MSL proteins interacts directly with autosomal targets, we examined MSL3 localization during early embryogenesis and discovered that this protein has broad autosomal binding before and after zygotic expression of MSL2. The chromatin binding of MSL3 is lost in early embryos lacking MSL1, suggesting interdependence. Taken together, these findings suggest that *roX* RNA and a subset of the MSL proteins bind throughout the genome during early development. The sensitivity of males to the loss of these factors reveals an underlying difference in autosomal heterochromatin in the sexes.

## Experimental Procedures

### Fly strains and culture

Flies were maintained at 25°C on cornmeal agar diet in a humidified incubator. *mof* ^*1*^, *mof* ^*2*^, *mle* ^*1*^ and *Jil-1* ^*z2*^ have been described [6–9]. The p[SUP or-P] insertion KV0020 is described [10]. The [*w* ^*+*^ actin-GAL4] [*w* ^*+*^-actin-GAL80^ts^] [*w* ^*+*^-UAS-*roX1*] third chromosome is from [11]. Mutant *roX1* transgenes *roX1* ^*+*^, *roX1* ^*AS*^, *roX1* ^*Δ6*^, *roX1* ^*Δ10*^, *roX1* ^*3'SLC*^ and *roX1* ^*7B*^ are described [12–14].

### Measurement of gene expression

Total RNA was prepared from 3 biological replicates of 50 3^rd^ instar males/genotype using the Trizol reagent (Invitrogen). One microgram of total RNA was reverse transcribed with ImProm-II reverse transcriptase following manufacturer recommendations (Promega). Duplicate reactions (5 μl of 1:20 template, 0.3 mM primers, and Applied Biosystems PCR master mix in a 25 μl volume) were amplified using an Mx3000P Real-Time PCR system (Stratagene). Genes selected for analysis were moderately and stably expressed during the third larval instar (genes and primer information in S1 Table). Calculations incorporate primer efficiency [15]. Initial studies of *mof* were normalized to the euchromatic autosomal gene *Dmn*. A second normalizing gene, *Ytr*, was added in subsequent studies of *mle* and *Jil-1*. Normalization to either single gene, or to both together, produced equivalent results.

### Embryo collection and chromatin preparation

Embryo fixation and chromatin preparation was performed as described [16]. In brief, 0.5 g of embryos was added to 9.2 ml cross linking buffer (50 mM HEPES, 1 mM EDTA, 0.5 mM EGTA, 100 mM NaCl, pH 7.6), 0.81 ml of 37% formaldehyde and 30 ml heptane. Samples were shaken vigorously for 20 min in a 50 ml conical tube, centrifuged for 1 min at 2,000 g and the supernatant discarded. Cross linking was stopped by vigorous shaking for 30 min in 25 ml PBS with 0.125 M glycine and 0.01% Triton X-100. Supernatant was discarded and fixed embryos processed for chromatin or flash frozen and stored at -80°C.

Five hundred mg of fixed embryos were washed in 10 ml of embryo wash buffer (10 mM HEPES, 1 mM EDTA, 0.5 mM EGTA, 0.1% sodium deoxycholate, 0.02% sodium azide, pH 7.6) and resuspended in 5 ml of sonication buffer (10 mM HEPES, 1 mM EDTA, 0.5 mM EGTA and 0.1% Triton X-100, pH 7.6) with proteinase inhibitor (Roche # 04693124001).

Sonication was performed for 70 cycles using a Fisher Model 500 Sonic Dismembrator and a 3.2 mm micro tip at 35% amplitude, 30 sec pulse and 59 sec cooling. Sonicated material was centrifuged 15 min at 16,000 g to remove debris. Supernatant was mixed with an equal volume 2X radioimmunoprecipitation (RIPA) buffer (2% Triton X-100, 280 mM NaCl, 20 mM Tris-HCl, 2 mM EDTA, 0.2% sodium dodecyl sulfate with protease inhibitor, pH 8.0) and precleared with blocked Protein G Agarose beads (Pierce Thermo Scientific # 20398). Aliquots of supernatant were flash frozen and stored at -80°C.

### Chromatin immunoprecipitation

Chromatin immunoprecipitation (ChIP) was performed essentially as described [16]. Two hundred fifty μl of chromatin, 250 μl RIPA buffer and 25 μl of anti-MSL3 antibody (gift of M. Kuroda) was gently mixed overnight and centrifuged at 16,000 g for 5 min. Supernatant was transferred to a new tube containing 40 μl of blocked protein G beads and mixed for 2 h. Beads were pelleted at 80 g and washed three times with low salt buffer (0.1% SDS, 1% Triton X-100, 2 mM EDTA, 20 mM Tris-Cl and 150 mM NaCl, pH 8.0), three times with high salt buffer (0.1% SDS, 1% Triton X-100, 2 mM EDTA, 20 mM Tris-Cl, 500 mM NaCl, pH 8.0), once with LiCl buffer (0.25 M LiCl, 1% NP-40, 1% sodium deoxycholate, 1 mM EDTA, 10 mM Tris-Cl, pH 8.0) and twice with Tris-EDTA (10 mM Tris, 1 mM EDTA, pH 8.0). Chromatin was eluted by two washes with 250 μl of freshly prepared elution buffer (1% SDS, 0.1 M monobasic NaHCO_3_, pH 8.0) at room temperature. Input was obtained by mixing 25 μl of pre-cleared chromatin and 475 μl elution buffer. Crosslinking was reversed by overnight incubation at 65°C in 0.2M NaCl, followed by RNase1 and Proteinase K digestion, phenol chloroform extraction and suspension in 50 μl distilled water.

### ChIP-qPCR analysis

Duplicate 20 μl reactions (10 μl of BioRad iTaq (# 172–5101), 4 μl of template, 4 μl 300 nM primer mix and 2 μl distilled water) were amplified using a Stratagene Mx3000P Real-Time PCR system. Enrichment was calculated by the ΔCt method [17, 18]. Input Ct values were corrected for dilution (Ct _[dilution corrected Input]_ = Ct _[Input]_—Log_2_ (Input Dilution Factor)). Input dilution factor is (fraction of the input chromatin saved)^-1^. Ct values from ChIP were normalized to the dilution corrected Input DNA Ct values (ΔCt _[normalized ChIP]_ = Ct _[ChIP]_—Ct _[dilution corrected Input]_). Normalized ΔCt values were converted to percent of Input (% Input = ((2 ^-ΔCt [normalized ChIP]^)*100). Genes and primers used for ChIP are presented in S2 Table.

## Results

### Analysis of heterochromatic gene expression in MSL mutants

Previous studies revealed that MSL1, MSL3 and *roX* RNA, but not MSL2, are necessary for full expression of heterochromatic genes in male flies [4]. This eliminates the possibility that the intact MSL complex, which requires MSL2, directly or indirectly regulates autosomal heterochromatic regions. Ectopic expression of MSL2 in females induces formation of MSL complexes that localize to both X chromosomes, but expression of MSL2 has no influence on PEV of reporters in autosomal heterochromatin [4, 5]. These observations suggest that, unlike some members of the MSL complex, loss of MSL2 affects only X-linked genes. To determine if the remaining MSL proteins were required for heterochromatic gene expression we used qRT PCR to measure a panel of genes in mutant male larvae.

**Fig 1:**
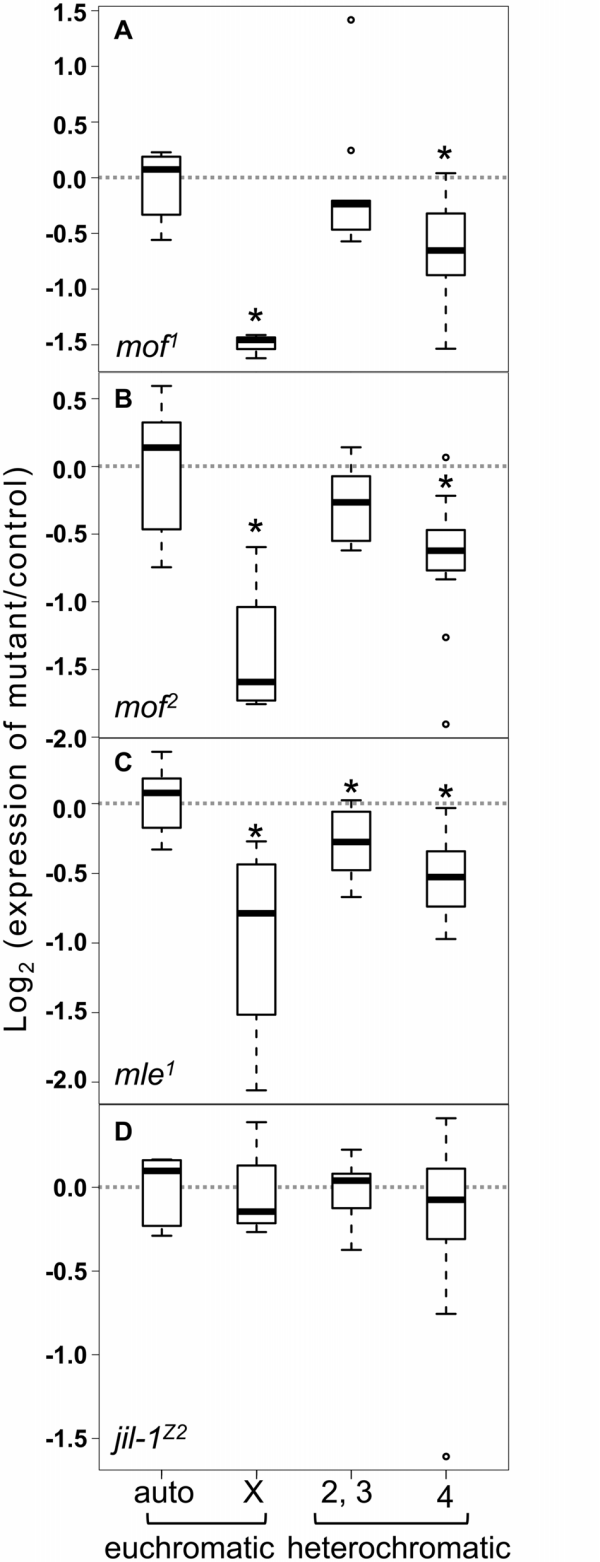
MLE is required for full expression of genes in heterochromatic regions. Expression was measured in **A)** *mof* ^*1*^, **B)** *mof* ^*2*^, **C)** *mle* ^*1*^ and **D)** *Jil-1* ^*z2*^ males. Controls were heterozygous (*mle/+; Jil-1/+*) or complemented (*mof*; [*mof* ^*+*^]) animals. Gene groups are euchromatic autosomal (ch 2 and 3, 6 genes analyzed, excepting *mle* ^*1*^ with 8 genes analyzed); X-linked (4 genes); heterochromatic (ch 2 and 3, 10 genes) and 4^th^-linked (14 genes). Genes and primers are presented in S1 Table. Expression of individual genes is presented in S1 Fig. The normalized Log_2_ expression ratio (mutant/control) is plotted (see methods for details). Box plots were generated using R. A Wilcoxon test comparing each gene group with euchromatic autosomal genes was used to determine *p-*values. Differences significant at a *p-*value of < 0.05 are marked with asterisks.

MOF is a histone acetyltransferase (HAT) that modifies lysine 16 on H4 (H4K16Ac), a mark enriched in compensated genes [19]. Mutant protein lacking HAT activity is present in *mof* ^*1*^ flies, but *mof* ^*2*^ mutants lack the protein altogether [8, 9]. While both alleles are male lethal, *mof* ^*1*^ females are healthy and fertile but *mof* ^*2*^ females are only weakly fertile, suggesting additional, non-catalytic functions [20]. We used quantitative reverse transcription PCR (qRT PCR) to measure gene expression in *mof* ^*1*^ and *mof* ^*2*^ male larvae (Fig 1A and 1B, S1A and S1B Fig). Expression was normalized to *Dmn*, a stably expressed euchromatic autosomal gene [4]. As expected, expression of X-linked genes is reduced in comparison to euchromatic autosomal genes in *mof* mutant males. Genes on the largely heterochromatic 4^th^ chromosome are also reduced. While heterochromatic genes on the 2^nd^ and 3^rd^ chromosomes are modestly reduced in expression, the sample size (10 genes) does not support a statistically significant conclusion.

MLE binds *roX* RNA and participates in assembly of the MSL complex, making it a likely cofactor in other *roX*-dependent processes [21, 22]. Expression of heterochromatic genes was examined in *mle* ^*1*^ male larvae (Fig 1C, S1C Fig). In addition to X-linked genes, heterochromatic genes on the 2^nd^, 3^rd^ and 4^th^ chromosomes displayed significant reductions relative to autosomal euchromatic genes in *mle* ^*1*^ males.

The kinase Jil-1 contributes to general chromatin organization and is essential in both sexes. However, hypomorphic Jil-1 mutations affect males more strongly than females, and, while Jil-1 binds interband regions throughout the genome, it is enriched on the male X chromosome [7, 23–25]. We measured expression in *Jil-1* ^*z2*^ animals, and found that none of the gene groups examined displayed changes in expression (Fig 1D, S1D Fig).

### Two *roX1* functions require different levels of RNA

To dissect the role of *roX1* in heterochromatic silencing we developed a genetic assay that takes advantage of suppression of PEV. Suppression of PEV requires simultaneous mutation of both *roX* genes and is limited to males [4, 5]. Flies were constructed with the partial loss of function *yw roX1* ^*ex33A*^ *roX2Δ* X chromosome, which supports 20% male eclosion. A variegating *y* ^*+*^ reporter on the second chromosome (p*[SUP or-P] KV0020;* hereafter KV20 [10]) was combined with a heat shock-inducible *roX1* expression system ([*w* ^*+*^-actin-GAL4] [*w* ^*+*^-actin-GAL80^ts^] [*w* ^*+*^-UAS-*roX1*] [11]). All transgenes comprising the inducible *roX1* expression system carry *w* ^*+*^, necessitating a switch to the *y* ^*+*^ marker of KV20 for detection of PEV. In an otherwise wild type male, KV20 produces an average of fewer than 50 pigmented spots on the abdomen (S2A Fig). Dramatic suppression of PEV in *yw roX1* ^*ex33A*^ *roX2Δ* males results in heavy pigmentation (~250 spots per abdomen).

To determine the critical time for *roX1* expression, flies were reared at 17°C and timed collections of embryos were heat shocked for 30 min at 37°C (S2B Fig). Heat shock was done before formation of the MSL complex (1.5–3 h), following zygotic MSL2 expression in males (4–6 h), mid embryogenesis (10–12 h), and during the final stages of cell division (12–14 h). To our surprise, non-heat shocked controls with inducible *roX1* displayed fully restored PEV (S2C Fig). In contrast, *yw roX1* ^*ex33A*^ *roX2Δ/Y;* KV20/+ males grown in parallel but lacking the inducible transgene continued to display suppression of PEV (S2C Fig, left). qRT PCR confirmed low *roX1* levels in larvae carrying the uninduced transgene (S3 Fig, Fig 3 of [11]). However, neither leaky expression nor a single 30 min heat shock during embryogenesis improved the survival of *yw roX1* ^*ex33A*^ *roX2Δ* males (S2C Fig inset). To determine if repeated heat shocks could rescue survival, we turned to the *roX1* ^*smc17A*^ *roX2Δ* combination, which is over 99% male lethal and provides a stringent test of *roX* function [14]. Induction of *roX1* expression in *roX1* ^*smc17A*^ *roX2Δ* males by daily 30 min heat shocks allowed 40% adult male eclosion (S4 Fig). This demonstrates that the transgene system is functional, but sustained *roX* expression is required during development to rescue male survival. We conclude that low levels of *roX1* expression from a leaky transgene are adequate to restore PEV, but not dosage compensation. The dosage compensation and heterochromatic integrity functions of *roX1* therefore require strikingly different RNA levels.

### *roX1* mutations differentially affect dosage compensation and PEV

**Fig 2:**
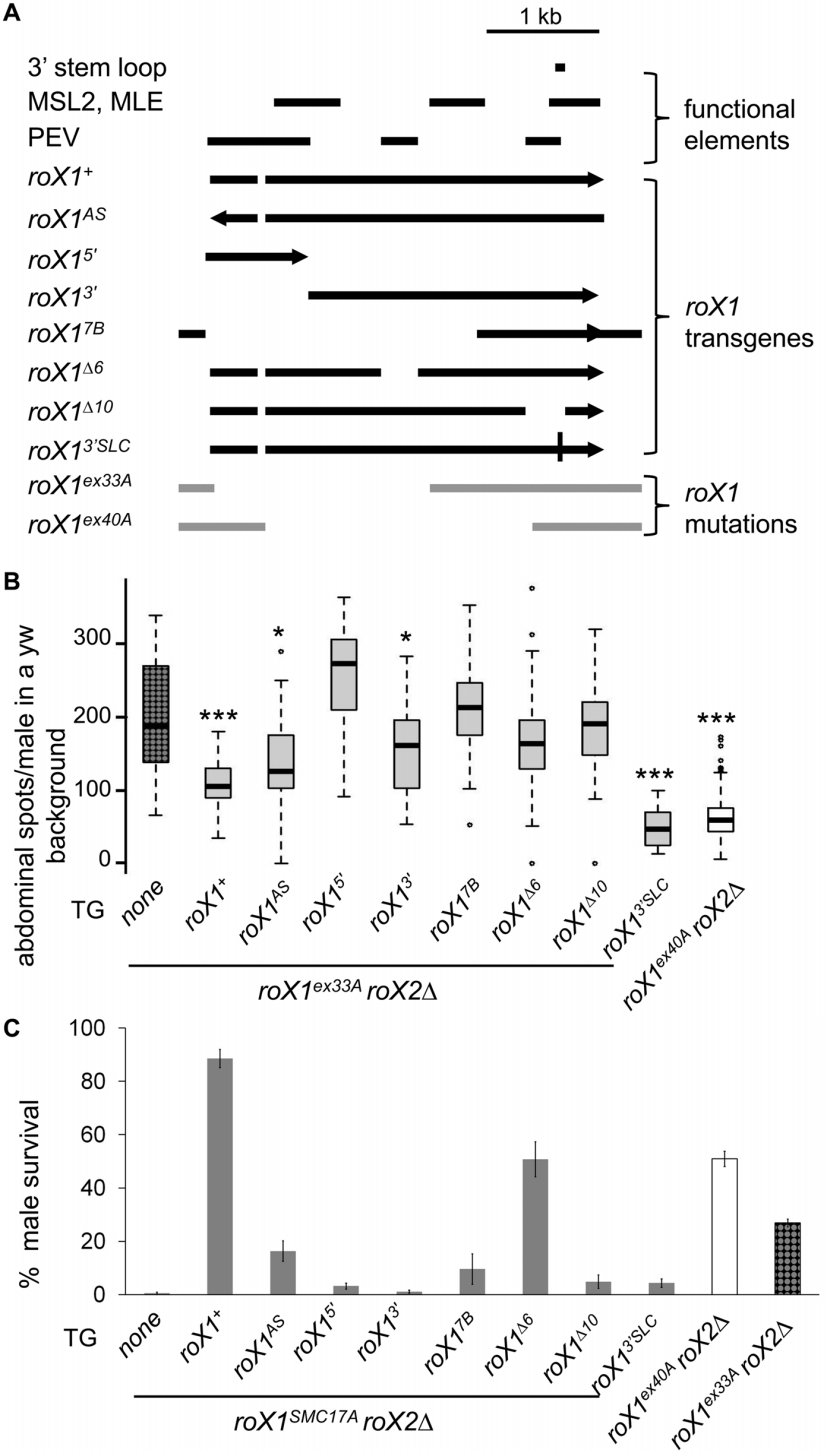
*roX1* mutations partially separate the dosage compensation and heterochromatic functions. **A)** *roX1* transgenes and mutations tested in this study. Functional regions of *roX1*, including the 3’ stem loop and sites of MSL2 and MLE binding (MSL2, MLE) are depicted. Regions that contribute to heterochromatic silencing in males are labeled PEV. *roX1* transgenes are driven by the hsp83 promoter. *roX1* ^*3'SLC*^ is a point mutation (black vertical line). Excision mutants *roX1* ^*ex33*^ and *roX1* ^*ex40A*^ are depicted in light gray. Mutations and transgenes are described [12–14] **B)** Suppression of PEV in in *yw roX1* ^*ex33A*^ *roX2Δ;* KV20/+ males is detected by increased abdominal pigmentation. Flies carry a single copy of *roX1* transgenes. *p-*values were generated by a Wilcoxon test. Each group was compared to *yw roX1* ^*ex33A*^ *roX2Δ;* KV20/+ (none). * *p*-value 0.05, *** *p*-value 0.0005. **C)** Rescue of *roX1* ^*SMC17A*^ *roX2Δ* males and survival of *roX1* ^*ex33A*^ *roX2Δ* and *roX1* ^*ex40A*^ *roX2Δ* males. Survival is calculated from the eclosion of sisters.

We then asked whether particular regions of *roX1* play differential roles in PEV and dosage compensation. To address this, deletions of *roX1* and mutated transgenes were tested in parallel for restoration of PEV in *roX1* ^*ex33A*^ *roX2Δ* males and rescue of *roX1* ^*SMC17A*^ *roX2Δ* survival. Adults are necessary for visualization of *y* ^*+*^ PEV. *roX1* ^*ex33A*^ allows ~ 20% adult male escapers, but is defective for heterochromatic silencing. In contrast, the extreme lethality of *roX1* ^*SMC17A*^ provides a sensitive background in which to detect rescue of dosage compensation by a *roX1* transgene. We generated *roX1* ^*ex33A*^ *roX2Δ;* KV20/+ males carrying each of the *roX1* transgenes depicted in Fig 2A. We also tested *roX1* ^*ex40A*^ *roX2Δ;* KV20/+ males, carrying a 2.3 kb internal *roX1* deletion that supports 50–100% male survival (Fig 2A). As expected, expression of an intact *roX1* cDNA (*roX1* ^*+*^) fully rescues male survival and restores PEV (Fig 2B and 2C). Antisense expression of the same cDNA (*roX1* ^*AS*^) largely restores PEV and provides a minor increase in survival, suggesting that internal promoters in the cloned fragment produce low levels of *roX1* transcript. The *roX1* gene has three main transcription start sites, two of which are internal to the cDNA used here [26]. As previously demonstrated, 5' and 3' *roX1* fragments are unable to rescue *roX1 roX2* males (Fig 2C *; roX1* ^*5’*^, *roX1* ^*3’*^; [12]). Neither fragment supports robust PEV, indicating that the heterochromatic *roX* function also requires both ends of the RNA (Fig 2B). A *roX1* transgene with a 2.4 kb internal deletion (*roX1* ^*7B*^) achieves minor rescue of male survival but is unable to restore PEV (Fig 2B and 2C). The *roX1* ^*Δ6*^ transgene, missing 325 bp, achieves good rescue of *roX1 roX2* male survival (Fig 2C). However, *roX1* ^*Δ6*^ did not support robust PEV, suggesting that the region removed is necessary for the heterochromatic function. *roX1* ^*Δ10*^ is deleted for 349 bp, removing a stem loop that is essential for dosage compensation [12]. Neither male lethality nor PEV are restored by *roX1* ^*Δ10*^, indicating that this region contributes to both functions. The *roX1* ^*3'SLC*^ transgene carries a point mutation that disrupts base pairing in the stem. As previously demonstrated, *roX1* ^*3'SLC*^ does not rescue male survival, but this transgene fully restores PEV (Fig 2B and 2C). Overlapping excisions *roX1* ^*ex33A*^ and *roX1* ^*ex40A*^ both remove the middle of *roX1*, including the region deleted in *roX1* ^*Δ6*^. Both mutants support considerable male survival. But while strong suppression of PEV is observed in *roX1* ^*ex33A*^ *roX2Δ* males, silencing remains intact in *roX1* ^*ex40A*^ *roX2Δ* males. As *roX1* ^*ex40A*^ contains 5’ regions that are excised from *roX1* ^*ex33A*^, we speculate that the 5’ end of *roX1* contains redundant elements that contribute to heterochromatic silencing. Taken together, these studies reveal that dosage compensation and heterochromatic silencing both require 5’ and 3’ elements of the *roX1* transcript. Both processes require a critical region at the 3’ end of *roX1*, however dosage compensation, but not heterochromatic silencing, requires intact base pairing of a stem loop within this region.

### MSL3 binds throughout the genome in early embryos

How *roX* RNA and a subset of MSL proteins contribute to heterochromatic silencing, and why this only occurs in males, remains unknown. All MSL proteins, excepting MSL2, are present at high levels in the oocyte. *roX1* is zygotically expressed before 2 h AEL, and, upon MSL2 expression at 3 h, a complex composed of MSL proteins and *roX1* RNA localizes to X chromatin [27–29]. The strikingly exclusive X chromosome binding of the MSL proteins in larvae suggests that autosomal binding during later developmental stages is unlikely. However, it is possible that maternally provisioned MSL proteins bind autosomal chromatin during early embryogenesis.

To test this idea we examined MSL3 localization in timed collections of control embryos (*yw* laboratory reference strain), and in embryos lacking maternal MSL1. Both MSL1 and MSL3 have been shown necessary for heterochromatic gene expression, suggesting that they might localize to affected regions [4]. MSL1 serves as the scaffold for assembly of the intact MSL complex, and is essential for all X chromosome binding [30]. Importantly, MSL3 levels remain high in *msl1* mutants, but MSL3 protein is no longer bound to chromatin [31, 32]. Zygotic expression of MSL1 initiates during stage 11 (5.2 to 7.2 h AEL), so early embryos from *msl1* mothers lack MSL1 entirely [28]. Staged collections of 1.5 to 3 h embryos (before MSL2 expression) and 4 to 6 h (after MSL2 expression and localization of the MSL complex to the male X chromosome) were generated and subjected to ChIP to detect MSL3 binding.

**Fig 3:**
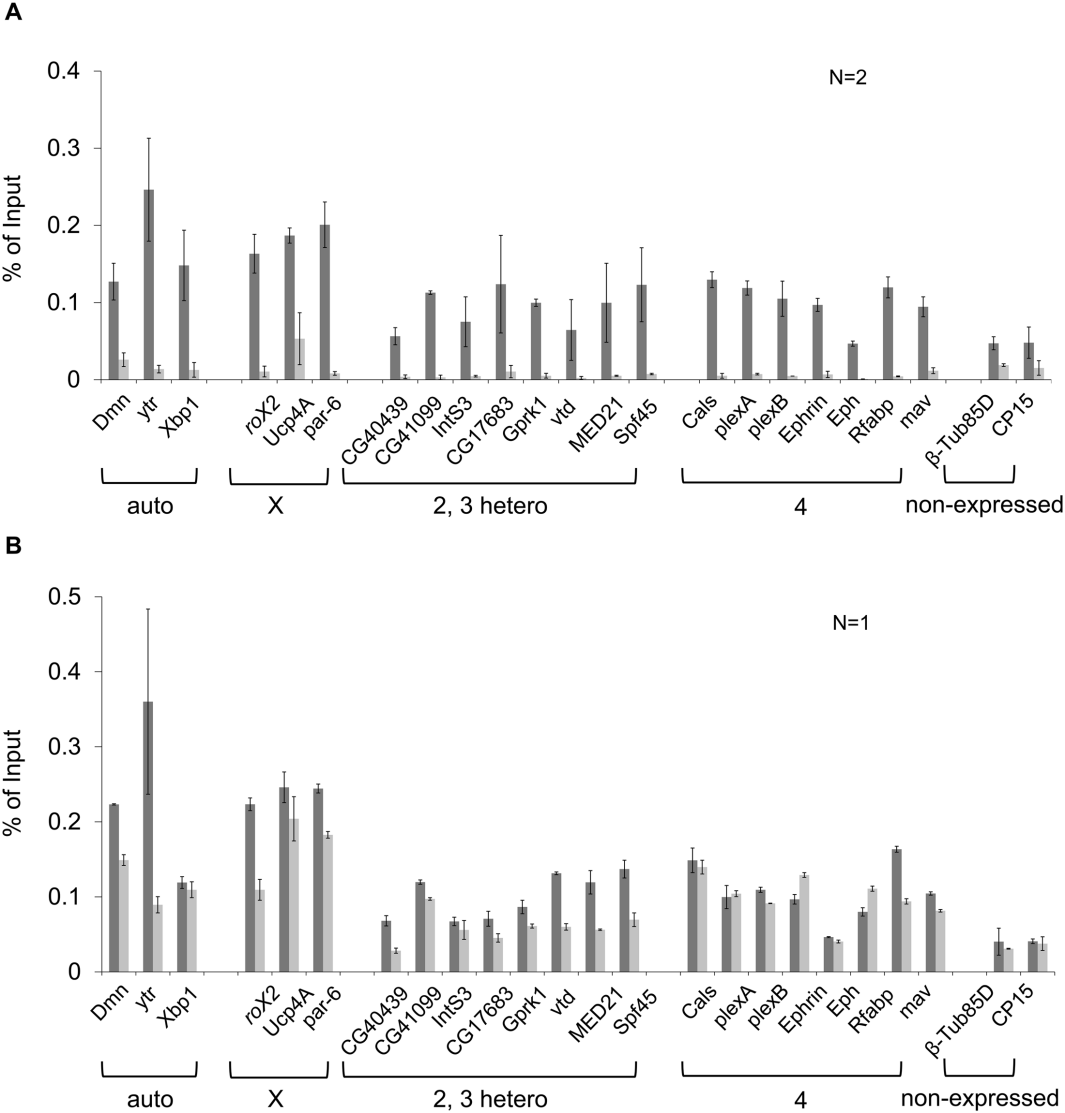
MSL3 binds throughout the genome of early embryos. Chromatin Immunoprecipitation was used to measure MSL3 enrichment at the indicated genes in embryos from wild type mothers (dark gray) and *msl1* ^*1*^/*msl1* ^*1*^ mothers (light gray). **A)** MSL3 enrichment in 1.5–3 h AEL embryos. Standard error is derived from two biological replicates with duplicate amplifications. **B)** MSL3 enrichment in 4–6 h AEL embryos. Duplicate amplifications of a single biological replicate are presented. Standard error is derived from duplicate amplifications. Primers are presented in S2 Table.

We decided to examine recruitment of MSL3 within genes for several reasons. The MSL complex is recruited to X chromosome gene bodies by binding of MSL3 to the cotranscriptional mark H3K36me3 [33]. As expression of heterochromatic genes is severely affected by mutation of *msl3*, it is possible that MSL3 is similarly recruited to active heterochromatic genes to prevent silencing. To test this we compared transcribed and non-expressed genes. Primer design is also simplified by selection of gene bodies, as these are unique sequences embedded in repetitive heterochromatin. ChIP-qPCR analysis of wild type 1.5 to 3 h embryos revealed widespread enrichment of MSL3 in the bodies of active euchromatic and heterochromatic genes on all chromosomes (Fig 3A, dark bars). Two genes with expression limited to male or female germ lines, β-Tub85D (β-Tubulin at 85D) and Cp15 (Chorion protein 15), served as non-expressed controls. High-shear ChIP-Seq revealed only minor MSL enrichment in these genes in S2 cells (S5A and S5B Fig [34]). Almost all active genes, regardless of chromosome or chromatin type, displayed higher MSL3 binding than the non-expressed controls. Interestingly, virtually all MSL3 enrichment was eliminated in embryos from *msl1/msl1* mothers, regardless of transcriptional status (Fig 3A, light bars). The dependence of MSL3 on MSL1 supports the idea that these proteins associate and localize at autosomal chromatin during early embryogenesis.

Between 4 and 6 h (stages 9 to mid-11) the intact MSL complex is assembled and recruited to the male X chromosome. Our expectation was that autosomal binding of MSL3 would be limited to early embryogenesis, prior to MSL2 expression at 3 h AEL. In contrast to this expectation, ChIP of control 4–6 h embryos revealed continued binding of MSL3 at autosomal genes, as well as enrichment at X-linked sites (Fig 3B, dark bars). The non-expressed controls showed very minor enrichment at this time point. Initiation of zygotic MSL1 expression in older embryos from this collection has largely restored MSL3 localization to X-linked genes in embryos from *msl1/msl1* mothers (Fig 3B, light bars). Interestingly, MSL3 binding at autosomal genes is also restored in 4–6 h embryos. We conclude that MSL3 is broadly localized in early embryos, and that this localization is dependent on MSL1. Restoration of autosomal binding in older embryos from *msl1/msl1* mothers indicates that autosomal localization persists after the onset of MSL2 expression. The distribution of MSL proteins during early development is consistent with the idea that a subcomplex of maternally provided MSL proteins binds broadly throughout the genome during early development.

## Discussion

A central question raised by this study is how factors known for their role in X chromosome dosage compensation also modulate autosomal heterochromatin. Although the MSL proteins were first identified by their role in X chromosome compensation [35], homologues of these proteins participate in chromatin organization, DNA repair, gene expression, cell metabolism and neural function throughout the eukaryotes [36–38]. Furthermore, flies contain a distinct complex, the Non-Sex specific Lethal (NSL) complex, containing MOF and the MSL orthologs NSL1, NSL2 and NSL3 [39, 40]. The essential NSL complex is broadly associated with promoters throughout the fly genome, where it acetylates multiple H4 residues [41, 42]. In light of the discovery that the MSL proteins represent an ancient lineage of chromatin regulators, it is unsurprising that members of this complex fulfill additional functions.

An alternative hypothesis for the dosage compensation of male X-linked genes proposes that the MSL proteins are general transcription regulators, and recruitment of these factors to the male X chromosome reduces autosomal gene expression, thus equalizing the X:A expression ratio [43, 44]. Arguing against this idea are ChIP studies finding that the MSL complex, and engaged RNA polymerase II, are increased within the bodies of compensated X-linked genes [45, 46]. In agreement with this, a study that normalized expression to genomic DNA concluded that compensation increases the expression of male X-linked genes [47]. Our current study now reveals that autosomal heterochromatic genes are indeed dependent on a subset of MSL proteins for full expression. However, native heterochromatic genes make up only 4% of autosomal genes, and their misregulation is not expected to compromise genome-wide expression studies normalized to autosomal expression.

Expression of heterochromatic genes is thought to involve mechanisms to overcome the repressive chromatin environment [2]. It is possible that a complex composed of *roX* RNA and a subset of MSL proteins participates in this process. This would explain why heterochromatic genes are particularly sensitive to the loss of these factors. Alternatively, it is possible that *roX* and MSL proteins participate in heterochromatin assembly. This would explain the simultaneous disruption of heterochromatic gene expression and suppression of PEV at transgene insertions. Heterochromatin assembly is first detected at 3–4 h AEL, a time when MSL3 is bound throughout the genome [48]. Intriguingly, studies from yeast identify a role for H3K4 and H4K16 acetylation in formation of heterochromatin [49, 50]. Active deacetylation of H4K16ac is necessary for spreading of chromatin-based silencing in yeast, demonstrating the need for a sequential and ordered series of histone modifications [50]. As MOF is responsible for the majority of H4K16ac in the fly, a MOF-containing complex could fulfill a similar role during heterochromatin formation. While our study found a significant effect of MOF in expression only on the X and 4^th^ chromosomes, it is possible that examination of a larger number of genes would reveal a more widespread autosomal effect. In *roX1 roX2* males the 4^th^ chromosome displays stronger suppression of PEV and more profound gene misregulation than do other heterochromatic regions [4]. This is consistent with the observation that heterochromatin on the 4^th^ chromosome is genetically and biochemically different from that on other chromosomes [51, 52].

Loss of *roX* RNA leads to misregulation of genes in distinct genomic regions, the dosage compensated X chromosome and autosomal heterochromatin. We find that the regulation of these two groups is, to some extent, genetically separable. MSL2, which binds *roX1* RNA and is an essential member of the dosage compensation complex, is not required for full expression of heterochromatic genes in males [4]. Ectopic expression of MSL2 in females induces formation of MSL complexes that localize to both X chromosomes, inducing inappropriate dosage compensation [53]. As would be expected from the lack of a role for MSL2 in autosomal heterochromatin in males, ectopic expression of this protein in females has no effect on PEV [5]. Elegant, high-resolution studies reveal that MLE and MSL2 bind essentially indistinguishable regions of *roX1* [21, 22]. Three prominent regions of MLE/MSL2 binding have been identified, one overlapping the 3’ stem loop. This stem loop incorporates a short “*roX* box” consensus sequence that is present in *D*. *melanogaster roX1* and *roX2*, and conserved in *roX* RNAs in related species [11, 12, 54, 55]. An experimentally supported explanation for the concurrence of MLE and MSL2 binding at the 3’ stem loop is that MLE, an ATP-dependent RNA/DNA helicase, remodels this structure to permit MSL2 binding [21, 56]. Our finding that disruption of this stem blocks dosage compensation but does not influence heterochromatic integrity is consistent with participation of *roX1* in two processes that differ in MSL2 involvement. However, a region surrounding the stem loop is required for the heterochromatic function of *roX1*, as *roX1* ^*Δ10*^, removing the stem loop and upstream regions, is deficient in both dosage compensation and heterochromatic silencing. Further differentiating these processes is the finding that low levels of *roX* RNA from a repressed transgene fully rescue heterochromatic silencing, but not dosage compensation.

An intriguing question raised by this study is why the sexes display differences in autosomal heterochromatin. The chromatin content of males and females are substantially different as XY males have a single X and a large, heterochromatic Y chromosome. We speculate that this has driven changes in how heterochromatin is established or maintained in one sex. A search for the genetic regulators of the sex difference in autosomal heterochromatin eliminated the Y chromosome and the conventional sex determination pathway, suggesting that the number of X chromosomes determines the sensitivity of autosomal heterochromatin to loss of *roX* activity [5]. Interestingly, the amount of pericentromeric X heterochromatin, rather than the euchromatic “numerator” elements, appears to be the critical factor. The recognition that heterochromatin displays differences in the sexes, and that a specific set of proteins are required for normal function of autosomal heterochromatin in males suggests a useful paradigm for the evolution of chromatin in response to genomic content.

## Supporting Information

S1 Fig Expression of individual genes in *mof*, *mle* and *Jil-1* mutant males. Larvae were homozygous for **A)** *mof* ^*1*^, **B)** *mof* ^*2*^, **C)** *mle* ^*1*^ and **D)** *Jil-1* ^*z2*^. Expression is relative to heterozygous (*mle*/+; *Jil-1*/+) or complemented (*mof*; [*mof* ^*+*^]) controls with otherwise identical genetic backgrounds. Values are derived from amplification of three biological replicates/genotype. The relative expression ratio (mutant/control) is normalized to *Dmn* and *ytr*, except for *mof*, where *Dmn* only is used. Averaged gene groups are presented at right. Genes and primers are presented in S1 Table.
(DOCX)

S2 Fig Position effect variegation (PEV) is a reporter for enforcement of heterochromatic silencing by *roX1*. **A)** The *roX1* ^*ex33A*^ *roX2Δ* chromosome is a suppressor of PEV. The *y* ^*+*^ marker in the KV20 insertion is partially silenced in control males carrying a wild-type *roX1* gene (left). Suppression of PEV in adult male escapers with a partial loss of function *roX1 roX2* chromosome (*yw roX1* ^*ex33A*^ *roX2Δ*; KV20/+, right) produces increased abdominal pigmentation (Konev *et al*. 2003). **B)** Assay to determine the critical time for *roX* in heterochromatin silencing. The repressed, heat shock inducible *roX1* transgene system ([UAS-*rox1* ^*18*^] [act-GAL4] [act-GAL80^ts^]) was introduced into *yw roX1* ^*ex33A*^ *roX2Δ*; KV20/+ flies [11]. Adult male escapers are scored for abdominal pigmentation. **C)** The repressed *roX1* transgene rescues PEV in the absence of heat shock. Staged collections of embryos reared at 17°C were heat shocked at 37°C for 30 min at times shown. The survival of adult males (right) and abdominal pigmentation (left) was determined. Control flies were not heat shocked or lack the inducible *roX1* transgene system (left). Box plots were generated using R.
(DOCX)

S3 Fig The inducible *roX1* transgene system is tightly regulated. *roX1* RNA was measured in the *yw* reference strain (wild type) and *roX1* ^*SMC17A*^ *roX2Δ* mutants with the *roX1* transgene system (*roX1* ^*SMC17A*^ *roX2Δ*; [UAS-*roX1*] [act-Gal4] [act-Gal80]). *roX1* ^*SMC17A*^ *roX2Δ* flies are deleted for qRT PCR primer binding sites. Expression is set to 1 in wild type flies and normalized to *Dmn* and *Ytr*. *roX1* transgene expression without heat shock is 5% of the heat shock induced expression.
(DOCX)

S4 Fig Rescue of male lethality by the inducible *roX1* transgene system. The inducible transgene partially rescues *roX1* ^*SMC17A*^ *roX2Δ* male survival after daily heat shocks. Developing embryos, larvae and pupae were heat shocked daily for 30 min at 37°C. Male survival is based on female emergence from the same vials. Full genotype: *roX1* ^*SMC17A*^ *roX2Δ*; [UAS-*roX1*] [act-Gal4] [act-Gal80].
(DOCX)

S5 Fig High-shear ChIP-Seq detects only minor enrichment of MSL proteins at non-expressed control genes [34]. **A)** B-Tubulin at 85D, expression limited to the male germ line. **B)** Chorion protein 15, expression limited to the female germ line. Enrichment (data set GSE37865) was visualized using the Integrated Genome Browser [57].
(DOCX)

S1 Table Primers used for gene expression qRT-PCR analysis. (DOCX)

S2 Table Primers used for ChIP-qPCR analysis. (DOCX)
