# Supplementary material for: Modulation of Heterochromatin by Male Specific Lethal Proteins and roX RNA in Drosophila melanogaster Males
Source: PLoS One. 2015 Oct 15;10(10):e0140259. doi: 10.1371/journal.pone.0140259 (PMC4607463; doi:10.1371/journal.pone.0140259)
Supplement: S1 Fig — Larvae were homozygous for A) mof 1, B) mof 2, C) mle 1 and D) Jil-1 z2. Expression is relative to heterozygous (mle/+; Jil-1/+) or complemented (mof; [mof +]) controls with otherwise identical genetic backgrounds. Values are derived from amplification of three biological replicates/genotype. The relative expression ratio (mutant/control) is normalized to Dmn and ytr, except for mof, where Dmn only is used. Averaged gene groups are presented at right. Genes and primers are presented in S1 Table. (DOCX) [file pone.0140259.s001.docx]

**S1 Fig.** **Expression of individual genes in *mof*, *mle* and *Jil-1* mutants.** Male larvae were homozygous for **A)** *mof^1^*, **B)** *mof^2^*, **C)** *mle^1^* and **D)** *Jil-1^z2^*. Expression is relative to heterozygous (*mle*/+; *Jil-1*/+) or complemented (*mof*; [*mof^+^*]) controls with otherwise identical genetic backgrounds. Values are derived from amplification of three biological replicates/genotype. The relative expression ratio (mutant/control) is normalized to *Dmn* and *ytr*, except for *mof,* where *Dmn* only is used. Averaged gene groups are presented at right. Genes and primers are presented in S1 Table.
