# Supplementary material for: Modulation of Heterochromatin by Male Specific Lethal Proteins and roX RNA in Drosophila melanogaster Males
Source: PLoS One. 2015 Oct 15;10(10):e0140259. doi: 10.1371/journal.pone.0140259 (PMC4607463; doi:10.1371/journal.pone.0140259)
Supplement: S2 Fig — A) The roX1 ex33A roX2Δ chromosome is a suppressor of PEV. The y + marker in the KV20 insertion is partially silenced in control males carrying a wild-type roX1 gene (left). Suppression of PEV in adult male escapers with a partial loss of function roX1 roX2 chromosome (yw roX1 ex33A roX2Δ; KV20/+, right) produces increased abdominal pigmentation (Konev et al. 2003). B) Assay to determine the critical time for roX in heterochromatin silencing. The repressed, heat shock inducible roX1 transgene system ([UAS-rox1 18] [act-GAL4] [act-GAL80ts]) was introduced into yw roX1 ex33A roX2Δ; KV20/+ flies [11]. Adult male escapers are scored for abdominal pigmentation. C) The repressed roX1 transgene rescues PEV in the absence of heat shock. Staged collections of embryos reared at 17°C were heat shocked at 37°C for 30 min at times shown. The survival of adult males (right) and abdominal pigmentation (left) was determined. Control flies were not heat shocked or lack the inducible roX1 transgene system (left). Box plots were generated using R. (DOCX) [file pone.0140259.s002.docx]

**S2 Fig. Position effect variegation (PEV) is a reporter for enforcement of heterochromatic silencing by *roX1*. A)** The *roX1^ex33A^roX2∆* chromosome is a suppressor of PEV. The *y^+^* marker in the KV20 insertion is partially silenced in control males carrying a wild-type *roX1* gene (left). Suppression of PEV in adult male escapers with a partial loss of function *roX1 roX2* chromosome (*yw roX1^ex33A^roX2∆*; KV20/+, right) produces increased abdominal pigmentation ([Konev *et al.* 2003](#_ENREF_2)). **B)** Assay to determine the critical time for *roX* in heterochromatin silencing. The repressed, heat shock inducible *roX1* transgene system ([UAS-*rox1^18^*] [act-GAL4] [act-GAL80^ts^]) was introduced into *yw roX1^ex33A^roX2∆*; KV20/+ flies ([10](#_ENREF_10)). Adult male escapers are scored for abdominal pigmentation. **C)**  The repressed *roX1* transgene rescues PEV in the absence of heat shock. Staged collections of embryos reared at 17˚C were heat shocked at 37˚C for 30 min at the indicated times. The survival of adult males (right) and abdominal pigmentation (left) was determined. Control flies were not heat shocked or lack the inducible *roX1* transgene system (left). Box plots were generated using R.
