# Supplementary material for: Modulation of Heterochromatin by Male Specific Lethal Proteins and roX RNA in Drosophila melanogaster Males
Source: PLoS One. 2015 Oct 15;10(10):e0140259. doi: 10.1371/journal.pone.0140259 (PMC4607463; doi:10.1371/journal.pone.0140259)
Supplement: S3 Fig — roX1 RNA was measured in the yw reference strain (wild type) and roX1 SMC17A roX2Δ mutants with the roX1 transgene system (roX1 SMC17A roX2Δ; [UAS-roX1] [act-Gal4] [act-Gal80]). roX1 SMC17A roX2Δ flies are deleted for qRT PCR primer binding sites. Expression is set to 1 in wild type flies and normalized to Dmn and Ytr. roX1 transgene expression without heat shock is 5% of the heat shock induced expression. (DOCX) [file pone.0140259.s003.docx]

**S3 Fig. The inducible *roX1* transgene system is regulated by heat shock.**

*roX1* RNA was measured in the *yw* reference strain (wild type) and *roX1^SMC17A^ roX2∆* mutants with the *roX1* transgene system (*roX1^SMC17A^ roX2∆*; [UAS-*roX1*] [act-Gal4] [act-Gal80]). *roX1^SMC17A^ roX2∆* flies are deleted for qRT PCR primer binding sites. Expression is set to 1 in wild type flies and normalized to *Dmn* and *Ytr*. *roX1* transgene expression without heat shock is 5% of the heat shock induced expression.
