# Supplementary material for: Modulation of Heterochromatin by Male Specific Lethal Proteins and roX RNA in Drosophila melanogaster Males
Source: PLoS One. 2015 Oct 15;10(10):e0140259. doi: 10.1371/journal.pone.0140259 (PMC4607463; doi:10.1371/journal.pone.0140259)
Supplement: S4 Fig — The inducible transgene partially rescues roX1 SMC17A roX2Δ male survival after daily heat shocks. Developing embryos, larvae and pupae were heat shocked daily for 30 min at 37°C. Male survival is based on female emergence from the same vials. Full genotype: roX1 SMC17A roX2Δ; [UAS-roX1] [act-Gal4] [act-Gal80]. (DOCX) [file pone.0140259.s004.docx]

**S4 Fig. Partial rescue of male lethality by the inducible *roX1* transgene system.**

Cultures were heat shocked daily for 30 min at 37°C. Male survival is based on female emergence from the same vials. Full genotype: *roX1^SMC17A^ roX2∆*; [UAS-*roX1*] [act-Gal4] [act-Gal80].
