# Supplementary material for: Modulation of Heterochromatin by Male Specific Lethal Proteins and roX RNA in Drosophila melanogaster Males
Source: PLoS One. 2015 Oct 15;10(10):e0140259. doi: 10.1371/journal.pone.0140259 (PMC4607463; doi:10.1371/journal.pone.0140259)
Supplement: S5 Fig — A) B-Tubulin at 85D, expression limited to the male germ line. B) Chorion protein 15, expression limited to the female germ line. Enrichment (data set GSE37865) was visualized using the Integrated Genome Browser [57]. (DOCX) [file pone.0140259.s005.docx]

A.

B.

**S5 Fig.** **High-shear ChIP-Seq in S2 cells detects only minor enrichment of MSL proteins at non-expressed control genes (**[**36**](#_ENREF_36)**)**. **A)** β-Tubulin at 85D (βTub85D), expression limited to the male germ line. **B)** Chorion protein 15 (Cp15), expression limited to the female germ line. Enrichment (data set GSE37865) was visualized using the Integrated Genome Browser ([63](#_ENREF_63)).
