# Supplementary material for: Modulation of Heterochromatin by Male Specific Lethal Proteins and roX RNA in Drosophila melanogaster Males
Source: PLoS One. 2015 Oct 15;10(10):e0140259. doi: 10.1371/journal.pone.0140259 (PMC4607463; doi:10.1371/journal.pone.0140259)
Supplement: S1 Table — (DOCX) [file pone.0140259.s006.docx]

**S1 Table. Primers used for gene expression qRT-PCR analysis.**

| Primer name | Primer orientation Left/Right | Primer sequence | Primer working  Conc.  (nM) | Primer  Efficiency (%) | Chromosome group |
| --- | --- | --- | --- | --- | --- |
| Dmn (CG8269) | Left | GACAAGTTGAGCCGCCTTAC | 300 | 98.5 | Autosomal euchromatic |
|  | Right | CTTGGTGCTTAGATGACGCA | 300 |  |  |
| Ytr (CG18426) | Left | ATTTTGGACCAGCACCACTC | 300 | 90.6 |  |
|  | Right | CAAAATCCCTGCAATTTCGT | 300 |  |  |
| LanB1 (CG7123) | Left | TCAACGAGCACCTGATTCAC | 300 | 94.5 |  |
|  | Right | GCAAATGGATGTTTCCCAAT | 300 |  |  |
| Xbp1(CG9415) | Left | GGGAGAGCAACTTTGACGAG | 300 | 97.1 |  |
|  | Right | GCCGGCCAAACTTAACAATA | 300 |  |  |
| Dip-B (CG9285) | Left | AGGATCACGCCAGAAGACTG | 300 | 92.8 |  |
|  | Right | AGTCACTGGGACGGAGAATG | 300 |  |  |
| Atp-α (CG5670) | Left | ACCCACACTGCTACACTCCC | 300 | 106.3 |  |
|  | Right | TCCTGGTTGCTCTTGTTGTG | 300 |  |  |
| GAPDH (CG9042) | Left | GCCCTGAACGGCAAGCT | 300 | 98.9 |  |
|  | Right | GTAAGATCCACAACGGAGACATTG | 300 |  |  |
| Bigmax (CG3350) | Left | CTCGGCGCACAATTCAGA | 400 | 97.6 |  |
|  | Right | CCCTTCTCCGCTCCTTGTAA | 300 |  |  |
| CKIIβ (CG15224) | Left | CCTGGTTCTGTGGACTTCGT | 300 | 98.4 | X-linked |
|  | Right | GTAGTCCTCATCCACCTCGC | 300 |  |  |
| Sgs4 (CG12181) | Left | GAAGGACCTGCTAACACCGA | 300 | 101.3 |  |
|  | Right | ATTTACACTTGGGTGCAGGC | 300 |  |  |
| SkpA (CG16983) | Left | CTAAAAGTCGACCAGGGCAC | 300 | 90.4 |  |
|  | Left | CCAGATAGTTCGCTGCCAAT | 300 |  |  |
| PpV (CG12217) | Right | TTGACCACCCATGAACTCAA | 300 | 94.2 |  |
|  | Left | GTGTTTGCTATGCTTGGGGT | 300 |  |  |
| GstT3 (CG1702) | Right | GACATCTTTGCAGCCTGTGA | 300 | 92.7 |  |
|  | Left | GCCCTGATCTTGGGGTACTT | 300 |  |  |
| Arc70/MED26 (CG1793) | Left | ATCGTACAACAACGAGCCCT | 300 | 86.4 | 4^th^ linked  4^th^ linked |
|  | Right | CAGCGTGAAAGAAACGTCAA | 300 |  |  |
| Cals (CG11059) | Left | AGTTTGTCAGCCCTCACCTT | 500 | 89.2 |  |
|  | Right | CTCCTATGCATTGCGACAGA | 500 |  |  |
| Ephrin (CG1862) | Left | TTGCAATTCTTGGCATTCAC | 300 | 95.2 |  |
|  | Right | CATAGAGGTCGCGGTGATTT | 300 |  |  |
| PlexA (CG11081) | Left | AAAGCAGCGATTGGCTTTTA | 500 | 86 |  |
|  | Right | GGCGCAGCTCTTATTCTGAC | 500 |  |  |
| JYalpha/CG17923 (CG45760) | Left | CCCCCTCCAGACTTAAAGGA | 300 | 94.1 |  |
|  | Right | CCATTCTTGTCCGTAGCCAT | 300 |  |  |
| RfaBp (CG11064) | Left | ACTGTCGCTGTCTTCCGATT | 300 | 88 |  |
|  | Right | GATTTTGCCTTTTTGTTCGC | 300 |  |  |
| Eph (CG1511) | Left | CTACCGTTTACCAGCTCCGA | 300 | 93.6 |  |
|  | Right | TTGCCAGCAATCCAACATTA | 300 |  |  |
| CG11077 | Left | GCCCTCGTATGGAACTGCTA | 300 | 87.5 |  |
|  | Right | CCGTATTACTTATGCGGGGA | 300 |  |  |
| Pho (CG17743) | Left | TCACGCAAAAGCAAAGAGAA | 300 | 88.6 |  |
|  | Right | ATTCAGCGTTTGAACAACCA | 300 |  |  |
| PlexB (CG17245) | Left | AACGGAACCACAAAAGATCG | 300 | 98.8 |  |
|  | Right | ATGTTACCGAGCGAACCAAC | 300 |  |  |
| Rad23 (CG1836) | Left | GCGGATAACGAAGACTTGGA | 300 | 99 |  |
|  | Right | TAGCCGTTCTATTGCGTCCT | 300 |  |  |
| Crk (FBgn0000375) | Left | AACATTAATGGGCAATGGGA | 300 | 92.6 |  |
|  | Right | CATCGACAAATTCAACGTGC | 300 |  |  |
| unc-13 (CG2999) | Left | GCGTTGGACGACTTAGCTTC | 300 | 99.9 |  |
|  | Right | CATGTCTCCAAGTTCTCGCA | 300 |  |  |
| Ank (CG1651) | Left | TGCAGAGTTTGGCACTCATC | 300 | 100.1 |  |
|  | Right | TCGCCATCTTTTTCAATTCC | 300 |  |  |
| Mav (CG1901) | Left | GATAAAATCGACGAGGCCAA | 300 | 104.4 |  |
|  | Right | TTTTCCTAGATCCTGGCCCT | 300 |  |  |
| lt (CG18028) | Left | CGAAGCTTCAACTGGCAATC | 300 | 94.5 | Chromosome 2&3 heterochromatic |
|  | Right | AGATAATGGTGCAAGCCCAC | 300 |  |  |
| Spf45 (CG17540) | Left | TCGAAAGTGCAATTAAAGCTG | 300 | 97.1 |  |
|  | Right | TAGTTGTAAAACCCCGCTCG | 300 |  |  |
| CG40439 | Left | TCTCGAGCATTGGGAGTTCT | 300 | 98.3 |  |
|  | Right | TGCCTTCCAAAGCTGCTATC | 300 |  |  |
| IntS3 (CG17665) | Left | GCAGAAGCAATAGCCGAATC | 300 | 97.2 |  |
|  | Right | AAACGCCTCCCAAGTGTATG | 300 |  |  |
| CG17683 | Left | AGGACTTTTTCAGCAAGGCA | 300 | 98 |  |
|  | Right | TGCTGAGCCTCACTAAGCAA | 300 |  |  |
| Gprk1 (CG40129) | Left | TCTAAAAGGCTTGGGTGCAT | 300 | 97.2 |  |
|  | Right | TGTAAACCTGGTGCCAATCA | 300 |  |  |
| CG12547 | Left | TTTTCCCGCAAAGATTGTTC | 300 | 96.3 |  |
|  | Right | CCCCGTATCAGCAATAGCAT | 300 |  |  |
| vtd/Rad21 (CG17436) | Left | CCCAGAACCACCTTCGTAGA | 300 | 94.7 |  |
|  | Right | GGACTGTGAAGGCATTGGTT | 300 |  |  |
| MED21 (CG17397) | Left | GGAAGTAGTGCAAAAAGGCG | 300 | 91.5 | Chromosome 2&3 heterochromatic |
|  | Right | TGAGCAATGCATTCCAAAGA | 300 |  |  |
| CG41099 | Left | GTGCGACATGGAATTGACAC | 300 | 87.6 |  |
|  | Right | CAATTGCGCGATGTAAAAGA | 300 |  |  |
