# Supplementary material for: Modulation of Heterochromatin by Male Specific Lethal Proteins and roX RNA in Drosophila melanogaster Males
Source: PLoS One. 2015 Oct 15;10(10):e0140259. doi: 10.1371/journal.pone.0140259 (PMC4607463; doi:10.1371/journal.pone.0140259)
Supplement: S2 Table — (DOCX) [file pone.0140259.s007.docx]

**S2 Table. Primers used for ChIP-qPCR analysis.**

| Primer name | Primer orientation  Left/Right | Primer sequence | Primer working Conc. (nM) | Chromosome group |
| --- | --- | --- | --- | --- |
| Dmn (CG8269) | Left | AGATTGAGCAGAAGCAGGGA | 300 | Autosomal euchromatic |
|  | Right | CAGCAGCTCCTTGTTGTTCA | 300 |  |
| Ytr (CG18426) | Left | CACAGCCTAGTGCAACGGTA | 300 |  |
|  | Right | CATGTACTCCAATTGCACCG | 300 |  |
| Xbp1(CG9415) | Left | CATACTGGGCTCTCCCACAT | 300 |  |
|  | Right | CTCGTCAAAGTTGCTCTCCC | 300 |  |
| GprK2 (CG17998) | Left | GTCGCTTCTTGGATGTCGAG | 300 |  |
|  | Right | CTGCGAGTTGTTGCTGTTGT | 300 |  |
| CG8173 | Left | AAATTAACCTCGCCCTGCTC | 300 | X-linked |
|  | Right | ACGGCGACCTTAAGTCCTTT | 300 |  |
| par-6 (CG5884) | Left | GGAACGATGAAACCGAGAAA | 300 |  |
|  | Right | ATAATGGCCGAGACTTGACG | 300 |  |
| Ucp4A (CG6492) | Left | CCCGTGAAGTTTGACTACGC | 300 |  |
|  | Right | TGGTCAGGTCCAAGGGATAG | 300 |  |
| roX2 (CR32665) | Left | GCAATTGGAACGAATTTGGA | 300 |  |
|  | Right | GAACTGCATGAATGCGAAAA | 300 |  |
| plexA (CG11081) | Left | AAAGCAGCGATTGGCTTTTA | 300 | 4^th^ linked |
|  | Right | TAGGATGGCGCAGCTCTTAT | 300 |  |
| Plex B (CG17245) | Left | TGAGAATACCATCGTGAGCG | 300 |  |
|  | Right | GGCTGTCCGGAAGATATTGA | 300 |  |
| Mav (CG1901) | Left | TTGGACTTGTTCAAAAGCGA | 300 |  |
|  | Right | TGTGTGGTGCGACTCTTTTC | 300 |  |
| Ephrin (CG1862) | Left | AGCTGTACGTGCCAGGAAGT | 300 |  |
|  | Right | TTTCGATGCATAGCTGGTTG | 300 |  |
| Cals (CG11059) | Left | TTCATCATCATTTTCGCTGG | 300 |  |
|  | Right | TTGAACTCCTGTTTGCTGGA | 300 |  |
| Rfabp (CG11064) | Left | AAGCGGAACATGAGGTTGAC | 300 |  |
|  | Right | GCGTTACCCACTTTCGATGT | 300 |  |
| Eph (CG1511) | Left | TGCTATAAGCGCCCTCAAGT | 300 |  |
|  | Right | TTGCTCGCTAATTGGAGCTT | 300 |  |
| CG41099 | Left | TCACTCCAGTCCAGCTGTTG | 300 | Chromosome 2&3 heterochromatic  Chromosome 2&3 heterochromatic |
|  | Right | AATCGTACTTCACGCTCGCT | 300 |  |
| CG17683 | Left | AAACCATGTCGAGGTTGAGC | 300 |  |
|  | Right | AACCTGTGAGGGCGTAATAAAA | 300 |  |
| IntS3 (CG17665) | Left | CATCTTTGCAATCAACAGCC | 300 |  |
|  | Right | TGCTCACGTCTCAAAGTTGTT | 300 |  |
| CG40439 | Left | CCATACTTGTGTGACGAACGA | 300 |  |
|  | Right | CCTGATTGGAAATTTTACTGACG | 300 |  |
| Spf45 (CG17540) | Left | ATGGATGGAAGGGCTTTACC | 300 |  |
|  | Right | GCCTTGCAGATCCAGAAGTT | 300 |  |
| MED21 (CG17397) | Left | TTACCTGATTAACGGTGTCTTGC | 300 |  |
|  | Right | TAAAATGGCGGATCGGCTTA | 300 |  |
| vtd /Rad21 (CG17436) | Left | GATCTGGTTGGCAGCACATT | 300 |  |
|  | Right | TTTGGTTGCAAAATCCCTTC | 300 |  |
| Gprk1 (CG40129) | Left | TGCATACTTACCCAAAACTTCG | 300 |  |
|  | Right | TGGAAAAAGAAGGCGAATTG | 300 |  |
| CG12547 | Left | TATCGTCATTCCATCGGTCA | 300 |  |
|  | Right | AAATGAAAAGGCTGGGACAA | 300 |  |
| β-Tub85D | Left | TCCCCTGCTAGCCTGTTTTG | 300 | Negative controls |
|  | Right | CACCGCCATTCAGGAGCTAT | 300 |  |
| Cp15 | Left | AGTTGTGGTAGTCCCCGTGA | 300 |  |
|  | Right | ACCATAACCTCCACGGTTGC | 300 |  |
